# Supplementary material for: Understanding the influence of self-concept on clinical decision-making among nurses: A cross-sectional study
Source: PLoS One. 2025 Aug 25;20(8):e0330905. doi: 10.1371/journal.pone.0330905 (PMC12377617; doi:10.1371/journal.pone.0330905)
Supplement: Attachment — Submitted filename: Response to Reviewers.pdf [file pone.0330905.s001.pdf]

Point-by-Point Response Table

Reviewer/Editor	Comment Number	Item	Comment	Response	Location in Manuscript
**Editor**	1	Style Requirements	Ensure manuscript meets PLOS ONE's formatting guidelines.	Thank you. The manuscript has been reformatted using PLOS ONE templates, including title page structure, section headings, and reference style.	Entire manuscript
**Editor**	2	Supplementary Files	Amend file type to 'Supporting Information'.	Thank you. All supplementary files (S1-S5 Tables, S1 Fig.) have been uploaded as 'Supporting Information' with legends listed after references.	End of manuscript (after References)
**Editor**	Q1	Conceptual Contribution	Limited theoretical innovation in established relationship.	We strengthened theoretical framing by integrating Bandura's self-efficacy theory and Benner's novice-to-expert model, emphasizing the Palestinian context's novelty.	Introduction (Theoretical Framework), Discussion
**Editor**	Q2	Title/Abstract	Descriptive rather than analytical.	Revised to highlight theoretical integration (Bandura/Benner) and contextual contribution	Abstract

				(Palestinian healthcare challenges), with effect sizes added.	
**Editor**	Q3	Introduction	Lacks conceptual framework.	Added dedicated Theoretical Framework section linking self-concept to CDM through Bandura and Benner's models.	Introduction
**Editor**	Q4	Methodology	Convenience sampling bias; no CFA; cross-sectional limitations.	Added CFA results (Methods), acknowledged sampling limitations with mitigation strategies (Methods), and proposed longitudinal designs (Discussion).	Methods (Pilot Study), Limitations
**Editor**	Q5	Results	Lacks depth in interpretation.	Added effect sizes (Cohen's d), confidence intervals, and subdomain analysis (leadership β=0.52).	Results, Table 4
**Editor**	Q6	Discussion	Overstates implications; ignores limitations.	Added measurement limitations (self-report bias), tempered causal claims, and proposed future studies with objective measures.	Limitations, Conclusion
**Reviewer #1**	1	Common Method Bias	Potential bias from self-report instruments.	Implemented procedural remedies: anonymous surveys, temporal separation of	Methods (Data Collection)

				instruments (24-48 hrs).	
**Reviewer #1**	2	Structural Equation Modeling	Suggested SEM for robust analysis.	Addressed via multiple regression with diagnostics; SEM recommended for future research.	Methods (Statistical Analysis)
**Reviewer #1**	3	Regression Model	Lacks theoretical variable selection.	Variables now justified via Bandura/Benner frameworks; added stress as moderator.	Introduction, Results
**Reviewer #1**	4	Discussion Depth	Superficial interpretation of Table 4.	Expanded regression interpretation (mediation effects, subgroup analysis).	Results, Discussion
**Reviewer #2**	1	Construct Definitions	Terms undefined (self-concept, CDM).	Added clear operational definitions in Abstract and Introduction.	Abstract, Introduction
**Reviewer #2**	2	Sampling	Justify convenience sampling.	Added logistical constraints (Palestinian healthcare system) and diversification strategies (11 hospitals).	Methods (Sampling)
**Reviewer #2**	3	Reliability Statistics	Missing Cronbach's alpha.	Reported α=0.89 (CDMNS) and α=0.90 (NSCQ) from study data.	Methods (Pilot Study)
**Reviewer #2**	4	Statistical Assumptions	Unreported diagnostics.	Added regression diagnostics (VIF, Durbin-Watson, Breusch-Pagan).	Methods (Statistical Analysis)

**Reviewer #2**	5	Language	Grammatical errors.	Corrected most instances (e.g., "decision making" → "decision-making"); recommend final proofread.	Throughout
**Reviewer #3**	1	Theoretical Grounding	Needs classical theories.	Integrated Bandura (1997) and Benner (1984) throughout.	Introduction, Discussion
**Reviewer #3**	2	Instrument Validation	Justify CDMNS/NSCQ use.	Added cultural adaptation details and CFA results (χ^2/df, CFI, RMSEA).	Methods (Measurements)
**Reviewer #3**	3	Methodological Details	Insufficient sampling info.	Added inclusion/exclusion criteria, participant flow diagram (Figure 1), hospital types.	Methods, Figure 1
**Reviewer #3**	4	Data Presentation	Tables need clarity.	Added descriptive captions, acronym definitions in footnotes (Tables 1-4).	All tables
**Reviewer #3**	5	Discussion	Repeats results.	Restructured to emphasize implications for education/policy (e.g., leadership training).	Discussion
**Reviewer #3**	6	Future Directions	Needs specific recommendations.	Added longitudinal designs, mixed-methods, cross-cultural comparisons.	Conclusion
