# Supplementary material for: Understanding the influence of self-concept on clinical decision-making among nurses: A cross-sectional study
Source: PLoS One. 2025 Aug 25;20(8):e0330905. doi: 10.1371/journal.pone.0330905 (PMC12377617; doi:10.1371/journal.pone.0330905)
Supplement: Attachment — Submitted filename: Response_to_Reviewers_auresp_2.pdf [file pone.0330905.s003.pdf]

Point-by-Point Response to Reviewers

Reviewer	Comment	Response	Changes Made
**Reviewer #1**	**1. Introduction lacks depth:** Needs stronger empirical foundation, cross-country studies, conceptual definitions, and measurement tool comparisons.	We thank the reviewer for this constructive feedback. The introduction has been expanded to: (1) Cite 8 additional studies from diverse contexts (Jordan, China, Australia), (2) Compare definitions of self-concept (Bandura vs. Cowin) and CDM (Jenkins vs. Tanner), and (3) Discuss measurement tools (CDMNS, NSCQ) and their validation.	Added 3 new paragraphs (lines 45–89) with references to Al Manaseer et al. (2025), Liu & Wang (2020), and Hoffmann & Murray (2019). Included a table comparing theoretical frameworks.
**Reviewer #1**	**2. Hypotheses lack theoretical support.**	We strengthened the theoretical basis by anchoring H1–H3 in Bandura's self-efficacy theory (H1) and Benner's novice-to-expert model (H2, H3), with citations to original works (Bandura, 1977; Benner, 1984).	Added explicit theoretical links to hypotheses (lines 210–225).
**Reviewer #1**	**3. Conclusions are superficial; methodology simplistic.**	We revised the conclusions to highlight: (1) Stress moderation as a novel finding, (2) Leadership self-concept's cultural specificity, and (3) Longitudinal research needs. Methodology now details countermeasures for common method bias (e.g., anonymization, instrument randomization).	Expanded conclusions (lines 540–560) and methods (lines 320–335).
**Reviewer #1**	**4. Weak logic; unclear focus.**	We reorganized the manuscript into 6 clear sections: Introduction, Literature Review, Methods, Results, Discussion, Conclusions. Added subheadings (e.g., "Cultural and Contextual Considerations") to improve flow.	Restructured entire manuscript; added 4 subheadings in Discussion.

**Reviewer #2**	All comments addressed.	We thank Reviewer #2 for their positive evaluation and confirmation that all concerns were resolved.	No further changes required.
**Reviewer #3**	All comments addressed; recommended for acceptance.	We appreciate Reviewer #3's thorough review and endorsement of our revisions.	No further changes required.
**Journal Requirements**	If the reviewer comments include a recommendation to cite specific previously published works, please review and evaluate these publications to determine whether they are relevant and should be cited. There is no requirement to cite these works unless the editor has indicated otherwise.	No reviewer comments included a recommendation to cite specific previously published works.	Confirmed no citation requests beyond general literature gaps.
