# Supplementary material for: Understanding the influence of self-concept on clinical decision-making among nurses: A cross-sectional study
Source: PLoS One. 2025 Aug 25;20(8):e0330905. doi: 10.1371/journal.pone.0330905 (PMC12377617; doi:10.1371/journal.pone.0330905)
Supplement: Attachment — Submitted filename: review to the manuscript_R1.doc [file pone.0330905.s002.doc]

1. The introduction should provide a more detailed explanation of why this issue is being studied and the significance of the research. It is necessary to add a solid empirical foundation. Currently, the literature review is insufficient, and incorporating studies from various countries would enhance its depth. Additionally, a conceptual definition should be included, highlighting the perspectives of different scholars. This comparison of how various researchers define key concepts, along with an exploration of measurement methods and authoritative measurement tools, will contribute to a richer research content.

2. The formulation of the research hypotheses appears to be inadequately supported by a theoretical basis.

3. The conclusions are somewhat superficial, and the academic rigor is insufficiently represented. The methodology is relatively simplistic, and the conclusions seemingly lack credibility.

4. The overall logic of the paper is relatively weak, and the focus of the content is not clearly defined. It is recommended to organize the material into 3-4 sections based on the study's logical framework (e.g., introduction, literature review, research methodology, analysis of results, discussion, and conclusions). Additionally, the study seemingly lacks depth.
